# Dynamic and Multi-Pharmacophore Modeling for Designing Polo-Box Domain Inhibitors

**DOI:** 10.1371/journal.pone.0101405

**Published:** 2014-07-18

**Authors:** Sugunadevi Sakkiah, Silvia Senese, Qianfan Yang, Keun Woo Lee, Jorge Z. Torres

**Affiliations:** 1 Department of Chemistry and Biochemistry, University of California Los Angeles, Los Angeles, California, United States of America; 2 Division of Applied Life Science (BK21 Program), Systems and Synthetic Agrobiotech Center (SSAC), Plant Molecular Biology and Biotechnology Research Center (PMBBRC), Research Institute of Natural Science (RINS), Gyeongsang National University (GNU), Jinju, South Korea; 3 Jonsson Comprehensive Cancer Center, University of California Los Angeles, Los Angeles, California, United States of America; 4 Molecular Biology Institute, University of California Los Angeles, Los Angeles, California, United States of America; Oak Ridge National Laboratory, United States of America

## Abstract

The polo-like kinase 1 (Plk1) is a critical regulator of cell division that is overexpressed in many types of tumors. Thus, a strategy in the treatment of cancer has been to target the kinase activity (ATPase domain) or substrate-binding domain (Polo-box Domain, PBD) of Plk1. However, only few synthetic small molecules have been identified that target the Plk1-PBD. Here, we have applied an integrative approach that combines pharmacophore modeling, molecular docking, virtual screening, and *in vitro* testing to discover novel Plk1-PBD inhibitors. Nine Plk1-PBD crystal structures were used to generate structure-based hypotheses. A common pharmacophore model (Hypo1) composed of five chemical features was selected from the 9 structure-based hypotheses and used for virtual screening of a drug-like database consisting of 159,757 compounds to identify novel Plk1-PBD inhibitors. The virtual screening technique revealed 9,327 compounds with a maximum fit value of 3 or greater, which were selected and subjected to molecular docking analyses. This approach yielded 93 compounds that made good interactions with critical residues within the Plk1-PBD active site. The testing of these 93 compounds *in vitro* for their ability to inhibit the Plk1-PBD, showed that many of these compounds had Plk1-PBD inhibitory activity and that compound Chemistry_28272 was the most potent Plk1-PBD inhibitor. Thus Chemistry_28272 and the other top compounds are novel Plk1-PBD inhibitors and could be used for the development of cancer therapeutics.

## Introduction

The Polo-like kinase (Plk) family of serine/threonine kinases are critical regulators of the cell cycle that are evolutionarily conserved from yeast to humans [1]. Plks are characterized by an N-terminal catalytic domain (kinase domain) and one or two C-terminal regions of similarity, termed polo-box domains (PBDs) [2]. PBDs are unique to Plks and are essential for regulating Plk phosphorylation activity through intramolecular interactions with the catalytic domain, binding to substrates and controlling Plk subcellular localization in a spatial-temporal manner [3]. These features make PBDs amenable to inhibition and are an ideal domain to explore the feasibility of inhibiting kinase phosphorylation activity by interfering with its intracellular localization and/or ability to bind substrates rather than targeting the conserved ATP binding site [4].

Humans express four Plk isoforms (Plk1-3 are closely related and Plk4 is distantly related) with apparently distinct expression patterns and physiological functions [5]. Plk1 is a mitotic kinase that regulates centrosome maturation and separation, mitotic exit and cytokinesis [6], Plk1 has been the focus of extensive studies due to its strong association with oncogenic transformation of human cells. Plk1 is overexpressed in many types of human cancers and plays a critical role in cellular proliferation from yeast to mammals [5]. Depletion or inhibition of Plk1 in cancer cells leads to mitotic arrest and subsequent apoptotic cell death [7]. Thus, Plk1 is an attractive target for anticancer therapy [8]. Over the years, efforts have been made to generate anti-Plk1 inhibitors, yielding several ATP-competitive inhibitors that inhibit Plk1 kinase activity [8]. These include BI2536 and GSK461364A, which are currently being evaluated for their anti-proliferative properties in clinical trials and numerous others that are in pre-clinical development [7]. However, their specificity and limited in vivo efficacy remain major concerns [9].

The Plk1-PBD plays a critical role in Plk1 subcellular localization, substrate binding and phosphorylation and is required for proper cell division [10]. Thus the Plk1-PBD has emerged as a candidate for therapeutic intervention and an alternative to targeting the Plk1 ATPase domain. The Plk1-PBD consists of two conserved polo boxes (PB1 and PB2), each of which exhibits folds based on a six-stranded β sandwich and an α helix, which associate to form a 12-stranded β sandwich domain [11]. Phosphoserine/phosphothreonine containing peptides comprising an S-(pT/pS)-(P/X) motif bind along a positively charged cleft formed between PB1 and PB2. The negatively charged phosphate groups of phospho-Ser/Thr residues interact with key amino acid residues at the PB1 and PB2 interface that include His538 and Lys540 from PB2 to form pivotal electrostatic interactions. The unique physical properties of the Plk1-PBD make it an attractive target for designing inhibitors with great specificity and potency. Indeed, *in vitro* screening efforts have already isolated small natural compounds, like Poloxin and Purpurogallin, and peptide-derived inhibitors like MQSpTPL that inhibit the Plk1-PBD from binding to substrate proteins [2], [7]. Although they are currently being evaluated for their antiproliferative properties *in vitro*, their lack of potency and issues associated with their solubility and delivery has limited their therapeutic potential [2], [7]. Additionally, to date there has been no attempts to generate a pharmacophore model of the Plk1-PBD-substrate interaction that would be instrumental for developing specific and potent inhibitors to this interaction.

Structure-based pharmacophore modeling has been successfully applied to designing of novel drugs with potent biological activity to many therapeutic areas. Structure-based pharmacophore models are generated by extracting the interaction between a protein and its ligand, which enables medicinal chemists to design new sets of ligands with the potential to be specific and potent drugs [12]. Even more powerful, pharmacophore models can be coupled to pharmacophore-based virtual screening and molecular docking studies to generate an integrative workflow for the discovery and development of novel inhibitors. Here, we have applied this type of integrative approach to better understand the Plk1-PBD-ligand interaction and to design novel Plk1-PBD inhibitors. Our study lends insight into the structural requirements crucial for inhibiting the Plk1-PBD and has discovered novel Plk1-PBD inhibitors, which can be used in designing and developing Plk1-PBD targeted therapies.

## Methods and Materials

### Selection of Plk1-PBD-ligand complex structures from the protein data bank

To generate structure-based Plk1-PBD pharmacophore models, we selected nine crystal structures, namely, 1Q4K [11], 1UMW [13], 3FVH [8], 3HIK [8], 3P34 [14], 3P37 [14], 4E9C [15], 4E9D [15], and 4HAB [16], based on their level of resolution and deposition date in the protein data bank (PDB, www.rcsb.org/pdb).

### Generation of receptor/structure based pharmacophore models

The Plk1-PBD structure-based pharmacophore models were derived from the critical interactions between the residues present in the active site of the receptor and the ligands. The biochemical data was used to identify the key residues that were important for substrate and/or inhibitor binding. To do this, LigandScout [17] was used to find the interactions between the inhibitors and critical residues in the Plk1-PBD binding site. It was also used for generating automatic hypotheses and visualization of pharmacophore models. The software utilized Plk1-PBD X-ray 3D crystal structures from PDB files to extract and interpret receptor-ligand interactions such as hydrogen bonds, charge transfers and hydrophobic regions within the macromolecular environment. Stepwise interpretation of the functional group patterns were performed for ligands: planar ring detection, assignment of functional group patterns, determination of the hybridization state and finally the assignment of Kekule pattern. Multiple chemical features and excluded volume spheres were detected and generated as structure-based pharmacophore models, which were used to screen small molecules for their ability to inhibit Plk1-PBD function. Subsequently the hypothesis generated by LigandScout (hypoedit) was subjected into Discovery Studio v 3.1 (DS, www.accelrys.com, chm file format) and converted into a suitable format for screening the multi-conformational 3D drug-like database.

### Drug-like database generation and virtual screening

Many drug candidates fail to perform well in pre-clinical and clinical settings. This is mainly due to their lack of potency against the intended drug target as well as pharmacokinetic and toxicity issues. Therefore, it is important for the drug design process to sort or remove the compounds that fail to satisfy the drug-like properties early on in the study. We initiated our study with a chemical database containing 159,757 diverse drug-like compounds that were subjected to energy minimization using dynamic simulations (DS). Next, we removed the compounds that did not pass the absorption, distribution, metabolism, excretion and toxicity (ADMET) properties [18] as well the rule of five properties [19]. The use of these filters resulted in 32,374 compounds that were used for virtual screening. The pharmacophore based virtual screening technique is a fast and cost effective computational tool to discover novel leads from database searches. In our study, the Hypo1 pharmacophore model was used for virtual screening of the drug-like database. While searching the pharmacophore against the database, we modified the parameters based on the number of chemical features present in Hypo1. The Fast Flexible search method from Ligand Pharmacophore Mapping implemented in DS was used to retrieve hits from the drug-like database. We changed the different Maximum Omitted Features option for Hypo1 to select compounds that matched a maximum of 4 chemical features. Database searching was performed based on feature mapping with every compound in the database and sorting according to highest fit value scores. The compounds that matched the atoms or functional groups and the geometric constraints between the small molecules and the query hypothesis were subjected to molecular docking studies.

### Molecular docking using LigandFit

Molecular docking is a computational tool used to predict protein-ligand interaction geometries and binding affinities. LigandFit [20] is a molecular docking program that was used to identify the suitable binding mode of the ligands within the Plk1-PBD and to predict their binding affinities. The crystal structure of the Plk1-PBD (PDB: 3P34) complex was retrieved from the PDB and used as the receptor protein. Initially, the Plk1-PBD was prepared for the docking process by removing all the water molecules and the CHARMm force field [21] was applied using the simulation tool. The protein active site is represented as a binding site for ligands that can be identified by applying two methods: (i) eraser algorithm which is based in the receptor shape and (ii) volume occupied by known ligand in the active site. Here, we employed the second strategy to identify the protein active site. The quality of the docking method was assessed by their ability to reproduce the binding mode of experimentally resolved protein-ligand complexes. To evaluate the accuracy of docking programs, co-crystal molecules were sketched and docked into the protein active site. The docked pose was superimposed on the co-crystal bound conformation to calculate the RMS deviation. An RMSD below 2 Å is generally considered a successful prediction. Herein a maximum of 10 poses for each ligand were selected and the RMS and the score threshold were set to 1.50 Å and 20 kcal mol-1, respectively. The scoring functions were based on the assumption that the binding affinity can be described as a sum of independent terms. The scoring functions included piecewise linear potential 1 (PLP1), piecewise linear potential 2 (PLP2), potential of mean force 04 (PMF04), dock score, Jain, Ligscore1, Ligscore2 and LUDI.

### Fluorescence polarization assay for evaluation of putative Plk1-PBD inhibitors

Plk1-PBD-substrate peptide binding assays was performed according to the protocol published by Reindl et al. [22]. The screening of the 93 candidate compounds was performed in 384-well plates (EK-30091) by incubating 67 nM recombinant human Plk1-PBD (Sigma SRP0360) with 8 nM fluorescein-labeled substrate peptide 5-carboxyfluorescein-GPMQSpTPLNG and 100 µM of each compound. After 1 hour incubation at room temperature, the fluorescence emission parallel (Int_parallel_) and perpendicular (Int_perpendicular_) to the plane of excitation at 535 nm was read on a 384 well plate reader (Tecan M1000). The fluorescence polarization (mP) was then calculated as: mP = (Int_parallel_ - Int_perpendicular_) / (Int_parallel_ + Int_perpendicular_) ×0.998×1000. The percent inhibition was calculated by normalizing the data to the DMSO only control. For calculation of IC_50_, we determined the fluorescence polarization of Plk1-PBD and the 5-carboxyfluorescein-peptide with a 12-point-2-fold-titration (from 49 nM to 100 µM) of each compound. The CDD (Collaborative Drug Discovery) software was used for generating IC_50_ values.

### Compound information

The top 93 compounds (at >90% purity) were acquired from commercial sources. See Table S1 for compound structure and vendor information.

## Results and Discussions

### Generation of a consensus structure-based pharmacophore model

**Figure 1:**
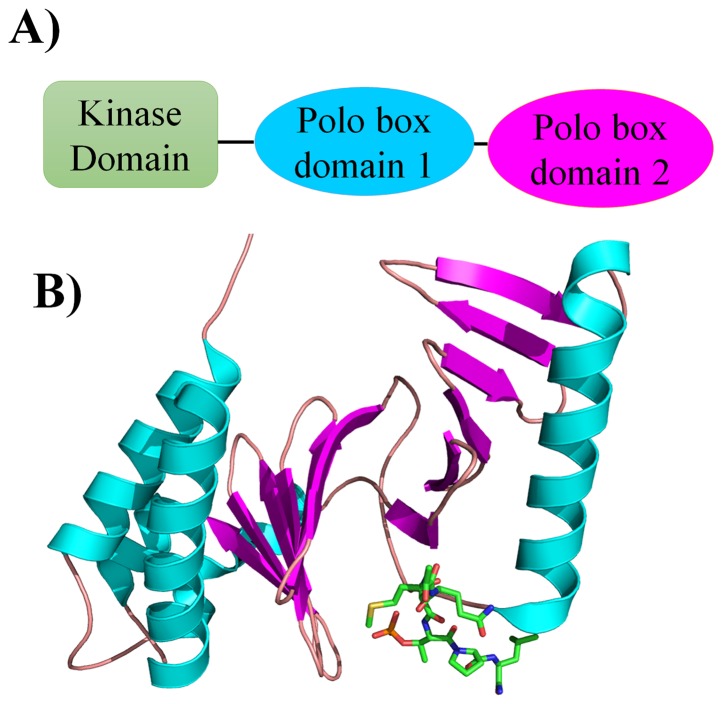
Plk1 kinase architecture. A) Plk1 has a modular domain structure with an N-terminal kinase domain and two C-terminal polo box motifs that make the polo-box domain (PBD). B) 3D co-crystal structure of the Plk1-PBD-ligand complex (PDB ID: 3P34). The Plk1-PBD and ligand are shown in secondary structure (ribbon and helix) and stick representation, respectively. Note the ligand-binding site is in a cleft formed by the two polo boxes.

**Figure 2:**
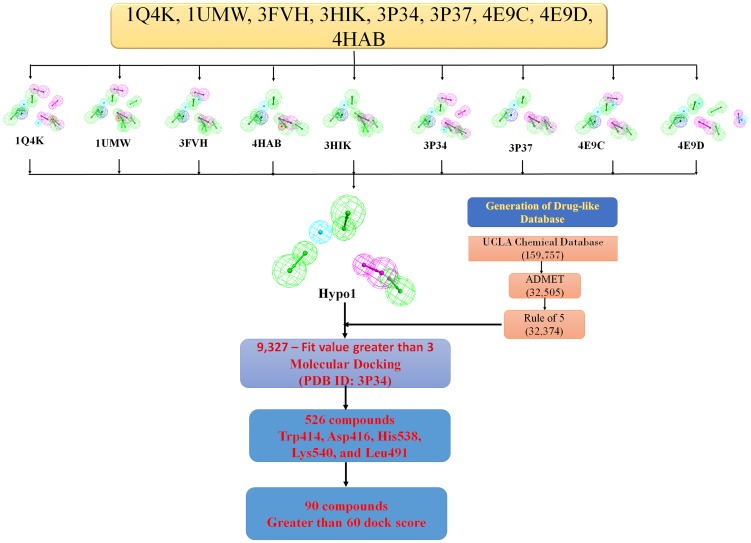
Integrative workflow for designing and virtual screening of polo-box domain inhibitors. Integrative workflow combines, pharmacophore modeling, generation of a drug-like database, virtual screening and molecular docking approaches to define the Plk1-PBD-ligand interaction and to identify Plk1-PBD inhibitors.

**Figure 3:**
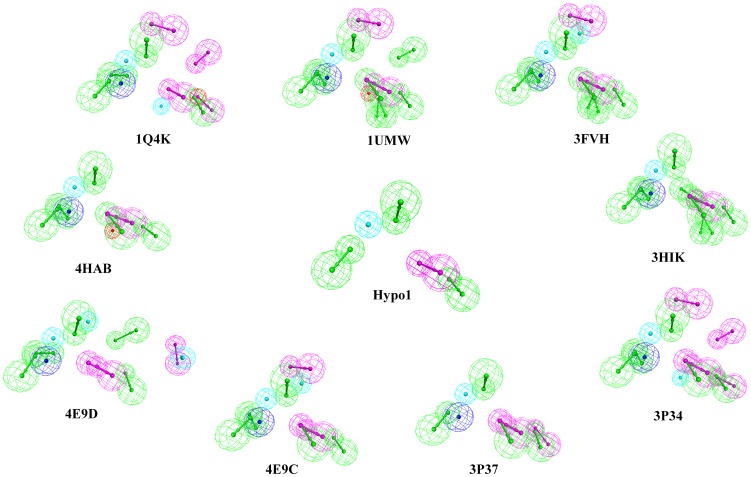
Generation of a common structure-based pharmacophore hypotheses using LigandScout. Hypo1 represents the five common chemical features present in all 9 hypotheses. Green, magenta, blue, red and cyan represents hydrogen bond acceptor, hydrogen bond donor, positive ionization, negative ionization and hydrophobic, respectively.

**Figure 4:**
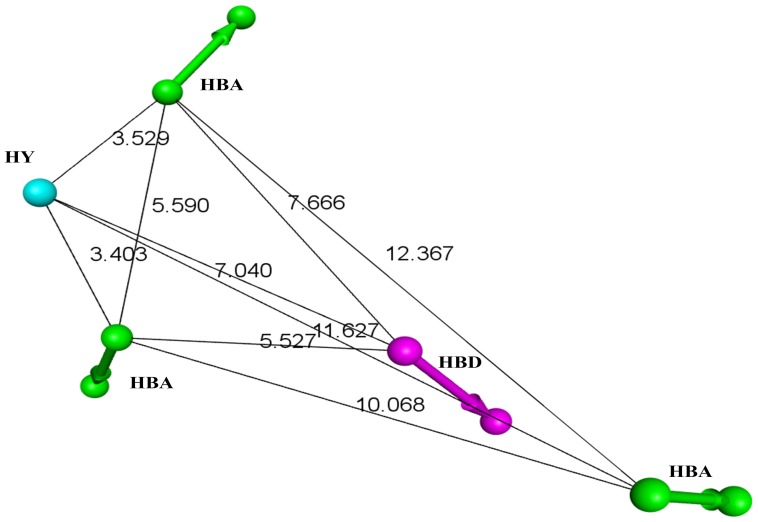
The five common chemical features in Hypo1 with their geometric constraints. Green, magenta and cyan represent hydrogen bond acceptor, hydrogen bond donor and hydrophobic, respectively. Edges represent distances in Angstroms.

The polo like kinase 1 (Plk1) has two potential sites of inhibition: its N-terminal ATPase domain and its C-terminal protein-binding domain, polo box domain (PBD) (Figure 1A). The Plk1-PBD is an attractive cancer target due to its unique structural properties that allow for the development of specific inhibitors targeting the protein-protein binding interface (Figure 1B). However, currently there is a lack of pharmacophore models describing the Plk1-PBD-ligand interaction, which would be beneficial for designing inhibitors. This prompted us to devise an integrated workflow for generating such a model (Figure 2). To do this, we first selected nine different Plk1-PBD X-ray crystal structures (1Q4K [11], 1UMW [13], 3FVH [8], 3HIK [8], 3P34 [14], 3P37 [14], 4E9C [15], 4E9D [15] and 4HAB [16]) from PDB as inputs for structure-based pharmacophore generation. For each Plk1-PBD-ligand complex, a pharmacophore model hypothesis was generated based on the critical interactions between the peptides and key residues in the active site of the Plk1-PBD with their specific geometric constraints (Figure 3). The common chemical features present in the all nine hypotheses were selected and named Hypo1 and the remaining chemical features were removed from further analyses (Figure 4). This approach reveled that five chemical features namely, 3-hydrogen bond acceptors, 1-hydrogen bond donor, and 1-hydrophobic were critical for inhibition of the Plk1-PBD.

### Generation of a drug-like database

Next, we sought to define the types of drug-like small molecules that could conform to the Hypo1 pharmacophore. However, to minimize downstream toxicity and efficacy issues, we were only interested in drug-like compounds with a strong potential for therapeutic use. Thus, we first established a drug-like database comprised of compounds that satisfied the criteria applied in ADMET and Rule of 5 that would be used for virtual screening. Hence an in house chemical library containing 159,757 diverse compounds was screened using ADMET and Rule of five. In ADMET, we mainly focused on the blood-brain barrier (BBB) permeability, solubility and absorption. The compounds were considered to have good drug-like properties only if they had values of 3, 3 and 0 for BBB permeability, solubility and absorption, respectively. After applying the ADMET criteria, 32,505 compounds showed good BBB permeability, solubility and absorption values. Subsequently these compounds were subjected to the Rule of 5, which states that the compounds are well absorbed only when they possess a logP less than 5, molecular weight less than 500 Da and fewer than 5 and 10 hydrogen bond donors and hydrogen bond acceptors, respectively. These criteria resulted in a database of 32,374 drug-like diverse compounds. This database was then used for subsequent virtual screening.

### Structure-based pharmacophore virtual screening

**Figure 5:**
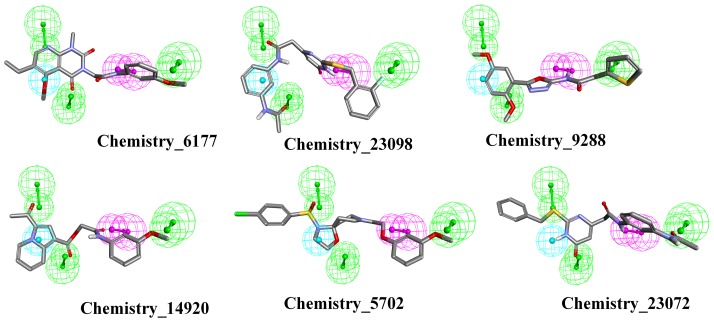
Hit compounds with a maximum fit value greater than 3. Representation of six compounds with a fit value greater than 3 identified through virtual screening. Note that compounds with diverse scaffolds are able to satisfy the geometric constraints of Hypo1 to form similar interactions. Green, magenta and cyan represents hydrogen bond acceptor, hydrogen bond donor and hydrophobic, respectively.

Virtual screening is an important computer-aided drug design method that is a cost-effective alternative to *in vitro* high-throughput screening. The Hypo1 hypothesis was used as a 3D query to screen the drug-like database of 32,374 compounds for compounds having 3 or more of the 5 Hypo1 features. This analysis resulted in 9,327 compounds with a fit value greater than 3. Examples of hit compounds are depicted in Figure 5.

### Molecular docking screening

To further analyze the selected compounds as potential Plk1-PBD inhibitors, they were subjected to molecular docking studies to determine their ability to bind within the Plk1-PBD and to study their critical interactions with the vital amino acids present in Plk1-PBD active site. To do this, we first analyzed the 23 Plk1-PBD-ligand complex structures deposited in the PDB to identify the critical residues making contacts with the ligands. Interestingly, His538 and Lys540 from PB2 were the only residues that contacted the phosphate groups present in the peptides directly. Trp414 and Leu491 also formed two important hydrogen bond interactions with the peptides. Although there were no conformational changes due to the peptide binding within the Plk1-PDB, there was a stretch in the β-sheet due to the hydrogen bond interaction between Asp416 and Met1 of the peptide [11]. Thus, all the complexes showed conserved hydrogen bond interactions with Trp414, Asp416, His538, Lys540 and Leu491 residues (Table 1). Consistently, previous mutagenic analyses of Trp414, His538 and Lys540 showed that these residues were critical for the binding of the Plk1-PBD to its substrates. For example, mutation of Lys540 to Met and/or His538 to Ala impaired Plk1-PBD binding to phosphorylated Cdc25 and Bub1 [13], [23]–[25]. Similarly, mutation of Trp414 to Phe abolished the association of Plk1-PBD with phosphorylated Cdc25 [3].

**Table 1:** Analyses of critical amino acids for Plk1-PBD inhibition from 23 Plk1-PBD-ligand co-crystal structures deposited in the protein data bank.

PDB ID	Trp414	Asp416	His538	Lys540	Leu491	His489	Arg516
3BZI	X	X	X	X	X	X	-
4HAB	X	X	X	X	X	X	X
4E9D	X	X	X	-	X	-	-
4E9C	X	X	X	X	X	X	-
3P37	X	X	X	X	X	-	-
3HIK	X	X	X	X	X	-	X
3FVH	X	X	X	X	X	X	X
4HY2	X	X	X	X	X	X	X
4HAB	X	X	X	X	X	X	-
3C5L	X	X	X	X	X	-	-
4DFW	X	X	X	X	X	-	-
3P2Z	X	X	X	X	X	X	X
3P36	X	X	X	X	X	X	X
3P35	X	X	X	X	X	X	-
3P34	X	X	X	X	X	X	-
4E67	X	X	X	X	X	-	-
1Q4K	X	X	X	X	X	X	-
3Q1I	X	X	X	X	X	-	X
1UMW	X	X	X	X	X	X	X
3HIK	X	X	X	X	X	-	X
3RQ7	X	X	X	X	X	-	X
2OJX	X	-	-	-	-	-	X
3BZI	X	X	X	X	X	X	-

X denotes hydrogen bonding with indicated amino acid.

**Figure 6:**
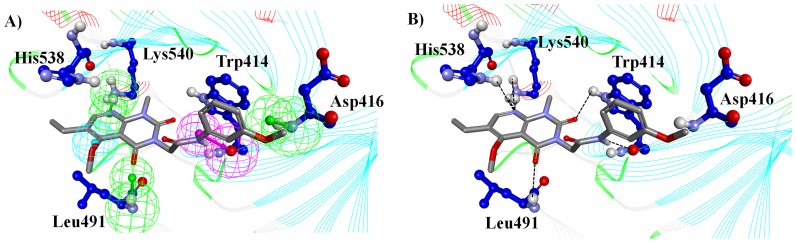
Docking of hit leads within the Plk1-PBD. A) Overlay of Hypo1 and the critical residues in the active site of Plk1-PDB with hit lead compound Chemistry_6177. B) Chemistry 6177 forms hydrogen bond interactions with key residues (Trp414, Asp416, His538, and Lys540) in the Plk1-PBD active site.

These five key residues were selected for screening the 9,327 drug-like compounds. To do this, the compounds were docked in the active site of Plk1-PBD and checked for good hydrogen bond interactions with the five key residues. This resulted in the identification of 526 compounds with good hydrogen bonding. Figure. 6 represents the binding orientation of one hit compound (Chemistry_6177) within the Plk1-PBD and also how well the compound fits into Hypo1. To further narrow down the candidate list we placed an extra filter based on the pose and a docking score greater than 60. This resulted in the identification of 93 high confidence compounds likely to inhibit the Plk1-PBD (Table S1). Interestingly, these compounds have diverse scaffolds that are able to satisfy the geometric constraints on Hypo1 to form similar interactions. Indicating that multiple avenues can be taken to develop therapeutics targeting the Plk1-PBD.

### Evaluation of putative Plk1-PBD inhibitors

**Figure 7:**
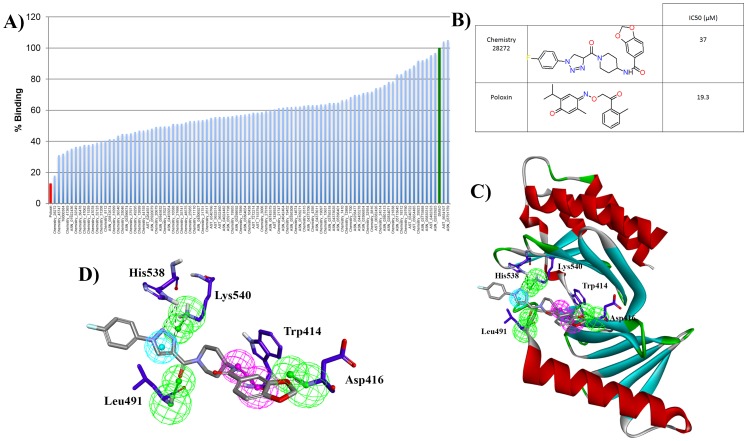
*In vitro* evaluation of the 93 putative Plk1-PBD inhibitors. A) Fluorescence polarization assay to measure the effect of DMSO (green bar), 100 µM Poloxin (red bar) or 100 µM of each of the 93 test compounds (blue bars) on the percent binding of Plk1-PBD to its substrate peptide 5-carboxyfluorescein-GPMQSpTPLNG. B) Chemical structure of Chemistry_28272 and Poloxin and their respective IC_50s_ for Plk1-PBD inhibition in fluorescence polarization assays described in A. C) Overlay of Hypo1 and the critical residues in the active site of the Plk1-PDB with hit lead compound Chemistry_28272. D) Zoomed view of ligand protein interaction showing that Chemistry_28272 forms hydrogen bond interactions with key residues (Trp414, Asp416, His538, and Lys540) in the Plk1-PBD active site.

To evaluate whether the top 93 high confidence compounds were indeed Plk1-PBD inhibitors, we acquired all 93 compounds and used a fluorescence polarization assay to test the ability of each compound to inhibit the binding of the Plk1-PBD to a fluorophore-labeled peptide that contained its optimal recognition motif, see Methods and Materials for complete details [22], [26]. In this assay, human Plk1-PBD (67 nM) was incubated with its substrate peptide 5-carboxyfluorescein-GPMQSpTPLNG (8 nM) in the presence of control DMSO, 100 µM Poloxin (a validated Plk1-PBD inhibitor [27]) or 100 µM of each of the 93 test compounds. The fluorescence polarization was calculated by analyzing the fluorescence emission at 535 nm with a multi-well plate reader. The percent inhibition was then calculated by normalizing the data to the DMSO control. Under these conditions, 28 out the 93 test compounds inhibited the Plk1-PBD to >50% inhibition (Figure 7A, Table S1). Interestingly, Chemistry_28272 showed almost complete inhibition of the Plk1-PBD similar to Poloxin (Figure 7A). To further analyze the potency of this top compound, we performed the same assay with a 12-point-2-fold-titration (from 49 nM to 100 µM) of Poloxin or Chemistry_28272. This analysis revealed that Poloxin had a half maximal inhibitory concentration (IC_50_) of 19.3 µM and Chemistry_28272 had an IC_50_ of 37 µM (Figure 7B). Figure 7C and 7D show the overlay of Hypo1 and the critical residues within the active site of the Plk1-PDB that make contact with Chemistry_28272, which indicate that Chemistry_28272 forms hydrogen bond interactions with key residues Trp414, Asp416, His538, and Lys540. These data indicate that 30% of the 93 compounds identified computationally as putative Plk1-PBD inhibitors had >50% Plk1-PBD inhibitory activity *in vitro* and that Chemistry_28272 represents the lead compound.

## Conclusions

The recent interest in developing inhibitors to the Plk1-PBD necessitates a comprehensive analysis of the Plk1-PBD-ligand interaction. Here, we have successfully developed a consensus structure-based pharmacophore model that describes the Plk1-PBD-ligand interaction. This structure-based pharmacophore model was integrated with virtual screening and molecular docking approaches to identify 93 potentially novel Plk1 inhibitors, which meet AMDET and Rule of five properties. The testing of these 93 compounds *in vitro*, with a Plk1-PBD-substrate binding assay, indicated that most of the 93 compounds had Plk1-PBD inhibitory activity and that Chemistry_28272 was the most potent compound with an IC_50_ of 37 µM. Chemistry_28272 represents a new class of Plk1-PBD inhibitors and could serve as a lead compound for further therapeutic development.

## Supporting Information

Table S1 Lists the top 93 compounds identified and tested *in vitro*, their chemical structures, vendor information, and experimental values from *in vitro* Plk1-PBD substrate peptide binding assays displayed as the percent binding.
(XLS)
